# Microfluidic capillary networks are more sensitive than ektacytometry to the decline of red blood cell deformability induced by storage

**DOI:** 10.1038/s41598-020-79710-3

**Published:** 2021-01-12

**Authors:** Nathaniel Z. Piety, Julianne Stutz, Nida Yilmaz, Hui Xia, Tatsuro Yoshida, Sergey S. Shevkoplyas

**Affiliations:** 1 Department of Biomedical Engineering, University of Houston, 3605 Cullen Blvd, Houston, TX 77204-5060 USA; 2 Hemanext, Lexington, MA USA; 3 Present Address: Department of Chemistry, Vanderbilt University, Nashville, TN USA

**Keywords:** Biomedical engineering, Blood flow, Haematological diseases

## Abstract

Ektacytometry has been the primary method for evaluating deformability of red blood cells (RBCs) in both research and clinical settings. This study was designed to test the hypothesis that the flow of RBCs through a network of microfluidic capillaries could provide a more sensitive assessment of the progressive impairment of RBC deformability during hypothermic storage than ektacytometry. RBC units (n = 9) were split in half, with one half stored under standard (normoxic) conditions and the other half stored hypoxically, for up to 6 weeks. RBC deformability was measured weekly using two microfluidic devices, an artificial microvascular network (AMVN) and a multiplexed microcapillary network (MMCN), and two commercially available ektacytometers (RheoScan-D and LORRCA). By week 6, the elongation indexes measured with RheoScan-D and LORRCA decreased by 5.8–7.1% (5.4–6.9% for hypoxic storage). Over the same storage duration, the AMVN perfusion rate declined by 27.5% (24.5% for hypoxic) and the MMCN perfusion rate declined by 49.0% (42.4% for hypoxic). Unlike ektacytometry, both AMVN and MMCN measurements showed statistically significant differences between the two conditions after 1 week of storage. RBC morphology deteriorated continuously with the fraction of irreversibly-damaged (spherical) cells increasing significantly faster for normoxic than for hypoxic storage. Consequently, the number of MMCN capillary plugging events and the time MMCN capillaries spent plugged was consistently lower for hypoxic than for normoxic storage. These data suggest that capillary networks are significantly more sensitive to both the overall storage-induced decline of RBC deformability, and to the differences between the two storage conditions, than ektacytometry.

## Introduction

To perform their physiological function, red blood cells (RBC) must undergo a wide range of deformations while traversing capillary networks as well as larger vessels^1,2^. An ability to sensitively detect changes in RBC deformability, and to accurately evaluate the potential impact of these changes on microvascular blood flow would be particularly useful in the field of transfusion medicine. Most blood transfusions involve RBCs that have been separated from donated whole blood, leukoreduced, suspended in a preservative solution, and stored in oxygen-permeable polyvinylchloride (PVC) bags at 2–6 °C for up to 6 weeks^3^. Prolonged hypothermic storage under these standard conditions is known to impair the biochemical and mechanical properties of RBCs^4–7^. Oxidative damage to the lipids and proteins comprising the RBC membrane and cytoskeleton is believed to be one of the primary causes of these storage lesions^8–11^. By the end of 6-week storage, each RBC unit consists of a highly heterogeneous mixture of cells in all stages of the storage-induced degradation^12–14^. Well-preserved RBCs remain flexible biconcave discs, while poorly-preserved and irreparably damaged RBCs become increasingly spiculated, spherical and less deformable as they accumulate damage^15^. As a result, the ability of stored RBCs to perfuse microvascular networks *in vitro*^16,17^, *ex vivo*^18^, and *in vivo*^19,20^ decreases, which may ultimately compromise the delivery of oxygen to tissues and vital organs of the recipient.

In laboratory and clinical practice RBC deformability is typically measured using ektacytometry^21,22^. To perform the measurement, a small sample of RBCs is suspended in a high-viscosity medium at a low hematocrit, and the suspension is subjected to a range of known shear stresses; the passage of elongated RBCs between the laser and the camera of the ektacytometer generates a characteristic laser diffraction pattern^23^. The relative lengths of the major and minor axes of the ellipse-shaped diffraction pattern are used to calculate the elongation index (EI), a dimensionless metric of deformability for the population of RBCs analyzed^21^. Importantly, the EI represents an aggregate of the mechanical properties of many thousands of RBCs that scattered light to create the diffraction pattern, rather than deformability of any individual RBCs that may be present in the sample. Therefore, ektacytometry may be relatively insensitive to small subpopulations of poorly deformable RBCs which are not numerous enough to significantly alter the aggregate diffraction pattern^24^. The use of samples with ultra-low hematocrit and un-physiologically high viscosity of the suspending medium may further limit the interpretation of the EI measurements^25^.

We have previously established the use of microfluidic network devices for measuring the ability of RBCs with impaired deformability to perfuse capillary networks^16,17,25^. The artificial microvascular network (AMVN) device evaluates the flow rate at which a sample of RBCs with physiological level of hematocrit and plasma viscosity is able to perfuse a network of capillary microchannels arranged in a pattern inspired by the layout of mesenteric microvasculature^16,26^. The multiplexed microcapillary network (MMCN) device comprises a network of narrowing microcapillaries arranged in a parallel array, and in addition to measuring the network perfusion rate also evaluates the frequency of intermittent plugging of its individual microcapillaries by poorly preserved RBCs that cannot deform sufficiently to traverse the 3 µm constriction, as well as the fraction of time the microcapillaries remain in a plugged state^17,27^. MMCN devices challenge individual RBCs to deform and pass through narrow constrictions thus enabling detection of poorly deformable cells within a population of otherwise normal RBCs, and AMVN devices enable quantification of the impact of these subpopulations of poorly deformable cells on overall perfusion rate under physiologically inspired conditions^17,25^.

The goal of this study was to compare the AMVN and MMCN perfusion and plugging measurements with the EI measurements by two commercially-available ektacytometers in the context of the storage-induced impairment of RBC deformability. We aimed to quantify the sensitivities of both methods to changes in RBC deformability over the course of hypothermic storage for individual RBC units. We also wanted to quantify the differences in RBC deformability measurements between RBC units with similar hematological parameters but different fractions of irreparably damaged RBCs. We hypothesized that AMVN and MMCN would be more sensitive than ektacytometry, because capillary network perfusion and plugging depend on the ability of individual RBCs to deform and pass through narrow constrictions while EI represents an aggregate measure of the entire RBC population. To enable these comparisons, we performed a split-unit study of RBCs stored under standard (normoxic) and hypoxic conditions over the course of 6-week hypothermic storage.

## Materials and methods

### RBC sample preparation

All experiments were performed in accordance with guidelines and regulations established by the University of Houston and the U.S. Department of Health and Human Services for the protection of human subjects. All experimental protocols involving human blood samples were approved by the University of Houston Institutional Review Board (Committee for the Protection of Human Subjects 1). All blood samples used in this study were purchased from Research Blood Components, LLC (Watertown, MA), which obtained written informed consent from all subjects. Whole blood was collected from healthy, consenting volunteers (n = 9) into citrate phosphate double dextrose (CP2D, Haemonetics, Briantree, MA). The blood was centrifuged (2000×*g* for 3 min) and mixed with 100 mL of AS-3 (Haemonetics, Briantree, MA). All units were leukoreduced by filtration (RC2D, Haemonetics, Briantree, MA) and split into two smaller units; one to be stored under standard (normoxic) and the other one under hypoxic conditions. RBC units to be stored under hypoxic conditions were deoxygenated by with a neonatal membrane oxygenator (Sorin D100, Sorin Group USA, Arvada, CO) using nitrogen sweep gas. RBCs were stored under both conditions in 150 mL capacity PVC blood bags (Teruflex T-150, Terumo Corp., Tokyo, Japan). Each hypoxically stored RBC unit was further packaged in an oxygen-impermeable bag (Rollprint Z, Addison, IL) containing 4 oxygen sorbent sachets (AGELESS SS-300, Mitsubishi Gas Chemical America, Inc., New York, NY). Processed RBC units were characterized at the start and after 6 weeks of storage by Hematology analyzer (Sysmex XE2100-D, Sysmex America) and cooximeter (ABL90, Radiometer USA). Units were stored in a blood bank refrigerator (Helmer iB111 i.Series, Helmer Scientific, Noblesville, IN) at 2–6 °C in blood bag holders (Blood Shoe, Genesis BPS, Ramsey, NJ) for 6 weeks.

Paired units of RBCs stored under normoxic and hypoxic conditions were evaluated side-by-side weekly. Each week, a 2 mL aliquot was withdrawn from each unit using the aseptic technique. After sampling a hypoxically stored unit, the oxygen sorbent sachets were replaced and the outer bag was resealed with a tabletop impulse heat sealer. The hematocrit of each RBC aliquot was adjusted to 40.0 ± 1.0% by adding a calculated volume of normal saline, and verified with an automated hematology analyzer (Medonic M-series, Boule Medical AB, Stockholm, Sweden). Samples with adjusted hematocrit were kept at 2–6 °C until use, and were allowed to reach room temperature and mixed by inversion before an experiment. Table 1 shows hematological parameters of each RBC unit measured at week 0 and week 6 of the study.

**Table 1:** Hematological parameters for RBCs stored under standard (normoxic) and hypoxic conditions at the beginning (week 0) and end (week 6) of hypothermic storage (n = 9). Values shown are mean ± standard deviation (range).

Parameter	Normoxic Storage		Hypoxic storage	
	Week 0	Week 6	Week 0	Week 6
Hct (%)	55.5 ± 2.8 (51.9–59.6)	54.9 ± 2.9 (51.1–58.8)	56.4 ± 2.6 (52.7–60.4)	55.5 ± 2.8 (51.5–59.7)
Hgb (g/dL)	18.1 ± 0.9 (16.9–19.5)	17.9 ± 0.9 (16.7–19.2)	18.4 ± 0.8 (17.2–19.7)	18.1 ± 0.9 (16.8–19.5)
MCV (fL)	93.8 ± 5.0 (88.2–101.9)	95.8 ± 4.1 (90.2–103.4)	93.5 ± 4.4 (88.2–101.1)	97.2 ± 4.1 (90.7–104.2)
MCHC (mg/dL)	32.3 ± 1.4 (30.5–35.3)	31.7 ± 0.7 (30.5–32.9)	32.4 ± 1.3 (30.5–35.3)	31.1 ± 0.9 (29.2–32.3)
RDW (%)	14.5 ± 1.6 (12.1–17.5)	15.3 ± 1.4 (12.7–17.2)	14.5 ± 1.5 (12.2–17.3)	14.9 ± 1.5 (12.3–17.2)
sO_2_ (%)	72.4 ± 12.2 (57.5–98.4)	97.7 ± 0.7 (96.8–98.8)	4.6 ± 1.2 (3.0–6.6)	0.9 ± 0.3 (0.4–1.4)
pCO_2_ (mmHg)	76.3 ± 12.2 (57.9–98.4)	64.6 ± 12.8 (32.7–78.9)	21.5 ± 20.9 (3.5–72.9)	41.8 ± 7.9 (31.8–59.4)
Hemolysis (%)	0.09 ± 0.04 (0.03–0.17)	0.30 ± 0.09 (0.17–0.46)	0.13 ± 0.05 (0.04–0.21)	0.28 ± 0.21 (0.14–0.85)

### Ektacytometry measurements

Ektacytometry measurements were performed according to the manufacturers’ specifications for each instrument. To perform the measurements using the RheoScan-D ektacytometer (RheoMeditech, Seoul, South Korea) the RBC sample was diluted to 1% by volume in polyvinylpyrrolidone (PVP) solution included in the RheoScan-D test kit (RSD-K02, RheoMeditech)^22,28,29^. A 500 μL volume of the diluted sample was then loaded into the disposable cartridge included in the RheoScan-D test kit, and the cartridge was inserted into the RheoScan-D instrument and evaluated automatically. Samples were evaluated at shear stresses ranging from 0.5 to 20.0 Pa. All steps necessary to perform the RheoScan-D measurement could be completed within ~ 5 min. To perform the measurements using the Maxsis Osmoscan Laser Optical Rotational Cell Analyzer (LORRCA, RR Mechatronics, Zwaag, the Netherlands) the RBC sample was diluted to 0.5% by volume in PVP solution included in the LORRCA reagents^21,29–31^. An 800 μL volume of the diluted sample was then loaded into the instrument and analyzed automatically. Samples were evaluated at shear stresses ranging from 0.3 to 30.0 Pa, at 37 °C and a camera gain level of 301. All steps necessary to perform the LORRCA measurement could be completed within ~ 5 min. For both ektacytometers, the elongation index (EI) was calculated as EI = (A − B)/(A + B), where A and B represent the major and minor axes of the ellipse-shaped RBC diffraction pattern^22^. RBCs suspended in PVP for both types of ektacytometry measurements cannot be recovered for additional analysis.

### Microfluidic device fabrication and measurements

**Figure 1:**
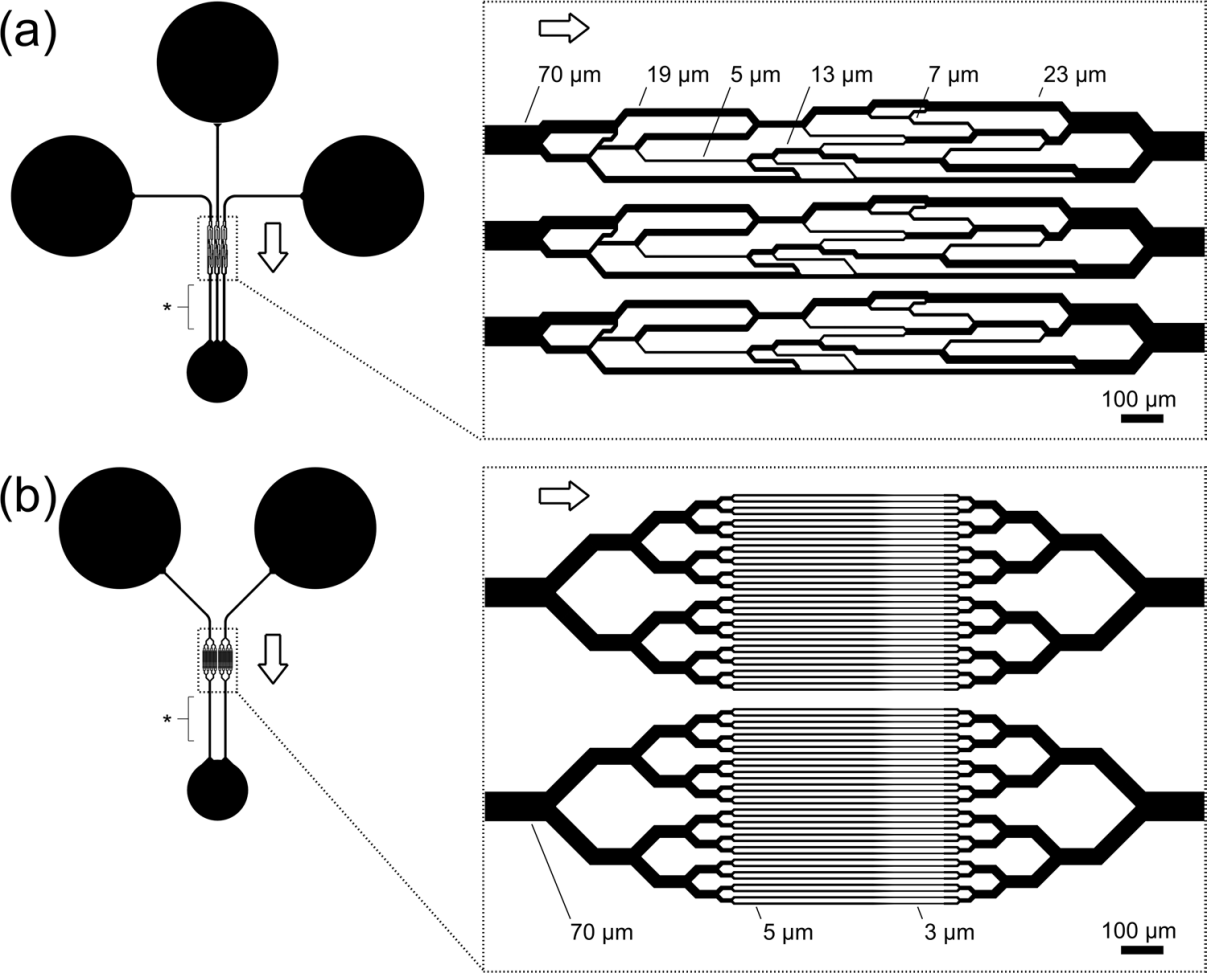
Schematic illustration of the microfluidic devices used to evaluate rheological properties of stored RBCs. (**a**) The artificial microvascular network (AMVN) device consisted of three parallel, identical networks of interconnected channels ranging in width from 70 µm to 5 µm. (**b**) The multiplexed microcapillary network (MMCN) device consisted of two parallel, identical networks of 32 microcapillaries, each narrowing in width from 5 µm to 3 µm. For both devices, each network unit had a separate inlet, and was connected to a shared outlet. All channels were approximately 5 µm deep. Arrows indicate the overall direction of flow in each device. Asterisks (*) indicate the sections of 70 µm wide outlet channels where images for perfusion rate measurements were acquired. Scale bars = 100 µm.

The design and fabrication of the artificial microvascular network (AMVN) microfluidic device (Fig. 1a) and the multiplexed microcapillary network (MMCN) microfluidic device (Fig. 1b) have been previously described in detail^16,17^. AMVN and MMCN devices were constructed from polydimethylsiloxane (PDMS) casted from molds of wafers fabricated via soft lithography^16,17^. Assembled microfluidic devices were incubated with a 1% solution of mPEG-silane (Laysan Bio, Inc., Arab, AL) in GASP buffer (1.3 mmol/L NaH_2_PO_4_, 9 mmol/L Na_2_HPO_4_, 140 mmol/L NaCl, 5.5 mmol/L glucose, 1% bovine serum albumin; osmolality 290 mmol/kg; pH 7.4) for at least 8 h to prevent non-specific adhesion of blood cells to channel walls. Devices were flushed with GASP buffer before use.

The method for measuring the perfusion rate in AMVN and MMCN devices has been previously described in detail^16,17^. Briefly, the pressure difference between the inlets and outlets of the AMVN and MMCN devices, used to drive flow through the devices, was controlled with an adjustable water column connected to device outlets via tubing and a barbed tube fitting (L420/410-6, Value Plastics, Fort Collins, CO). To perform the perfusion rate measurements, 25 μL of RBC samples with adjusted hematocrit were added to each device inlet and allowed to fully perfuse the microfluidic network (confirmed visually via microscope). Images of the 70 µm downstream ‘venule’ channels were captured using an inverted bright-field microscope (IX71, Olympus America Inc., Center Valley, PA) equipped with a high-speed digital camera (Flea3, Point Grey Research, Inc., Richmond, Canada). Image capture was initiated with driving pressure set to 0 cmH_2_O, then after 30 s the driving pressure was set to −20 cmH_2_O, and bursts of 10 images at 150 fps were collected at 10 s intervals for the duration of each 6-min measurement. The −20 cmH_2_O driving pressure was selected to approximate physiological microcapillary flow conditions. Image sets were analyzed offline using a custom image analysis algorithm implemented in MATLAB (The Math Works Inc., Natick, MA, USA), as previously described^16^. The procedure for measuring plugging of MMCN capillaries was similar to the perfusion rate measurements, only the images were captured of the capillary portion of the device (750 images at 150 fps). Capillary plugging was defined as a decrease in RBC flow rate within a MMCN microcapillary below a cutoff value of the mean RBC flow rate at week 0, minus two standard deviations, as previously described^17^.

### Morphology measurements

Images of RBCs for morphological analysis were obtained using a simple microfluidic device, as previously described^13^. For each morphology measurement, a set of 200 images (each depicting an individual RBC) were classified visually by a blinded expert scorer using a guide of representative images for each morphology class (Fig. 5d). Each RBC was classified as either a discocyte (D), an echinocyte I (E1), an echinocyte II (E2), an echinocyte III (E3), a sphero-echinocyte (SE), a spherocyte (S), a stomatocyte I (ST1) or a stomatocyte II (ST2). Morphology Index (MI) was calculated as previously described: MI = [− 2(#ST2) − 1(#ST1) + 0(#D) + 1(#E1) + 2(#E2) + 3(#E3) + 4(#SE) + 5(#S)] / #total^32,33^.

### Statistical analysis

Statistical analysis was performed using built-in functions of MATLAB 2014b Statistics Toolbox (The Math Works Inc., Natick, MA). A paired, two-tailed *t* test was used for the comparison between two groups. A *p* value of ˂ 0.05 was considered statistically significant.

## Results

### Measurements of RBC deformability using ektacytometry

**Figure 2:**
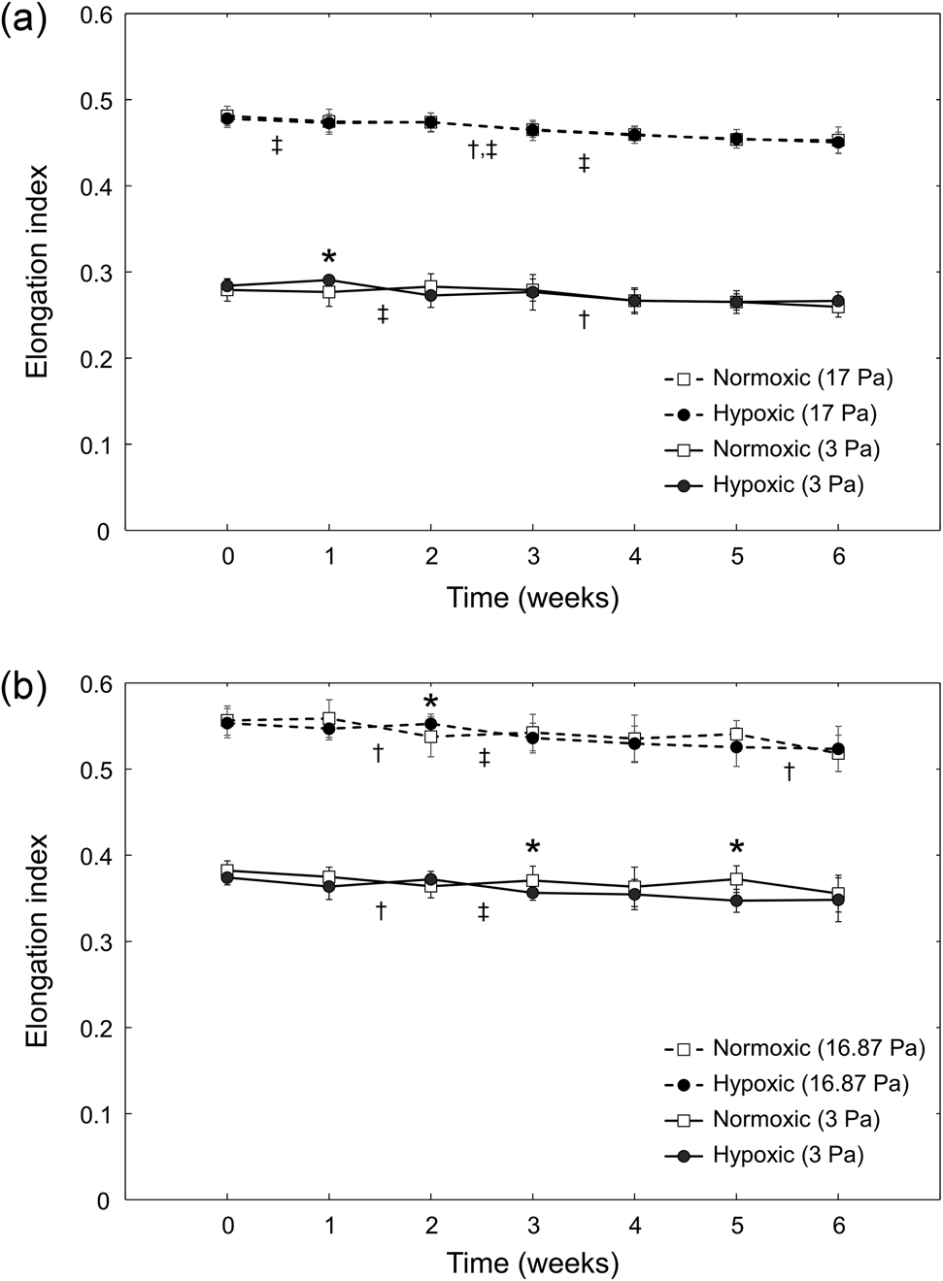
Elongation index (EI) measured using ektacytometry for RBCs stored under normoxic and hypoxic conditions over the course of 6-week hypothermic storage. (**a**) EI measured using RheoScan-D ektacytometer for two levels of shear stress (3 Pa and 17 Pa). Values shown are mean ± standard deviation (n = 5). (**b**) EI measured using LORRCA ektacytometer for two levels of shear stress (3 Pa and 16.87 Pa). Values shown are mean ± standard deviation (n = 4). Statistically significant differences (*p* < 0.05) between normoxic and hypoxic storage of the same duration are marked by an asterisk (*). Statistically significant differences (*p* < 0.05) between subsequent weeks of storage are marked by a dagger (†) for normoxic, and a double dagger (‡) for hypoxic storage.

Figure 2 shows the EI values measured using the two commercially-available ektacytometers (RheoScan-D and LORRCA). The EI for normoxically stored RBCs (normoxRBCs) and hypoxically stored RBCs (hypoxRBCs), as measured at low (3 Pa) and high (~ 17 Pa) shear stresses, decreased progressively over the course of 6-week hypothermic storage. Between week 0 and week 6, the EI measured with RheoScan-D decreased by 7.1% for normoxRBCs and by 6.2% for hypoxRBCs at 3 Pa, and decreased by 5.8% for normoxRBCs and by 5.8% for hypoxRBCs at 17 Pa (Fig. 2a). RheoScan-D measurements at 3 Pa showed a statistically significant decrease in EI compared to week 0 after 4 weeks for both types of storage. RheoScan-D measurements at 17 Pa showed a statistically significant decrease in EI after 3 weeks for normoxRBCs and after 1 week for hypoxRBCs. Over the same storage duration, the EI measured with LORRCA decreased by 7.0% for normoxRBCs and by 6.9% for hypoxRBCs at 3 Pa, and decreased by 6.9% for normoxRBCs and by 5.4% for hypoxRBCs at 16.87 Pa (Fig. 2b). LORRCA measurements at 3 Pa showed a statistically significant decrease in EI compared to week 0 after 2 weeks for normoxic storage, and after 3 weeks for hypoxic storage. At 16.87 Pa, LORRCA measurements showed a statistically significant decrease in EI after 3 weeks for both types of storage. There was no consistent, statistically significant difference between the EI of RBCs stored under normoxic or hypoxic conditions at either low or high shear stresses (Fig. 2).

### Perfusion rates in the microfluidic capillary networks

**Figure 3:**
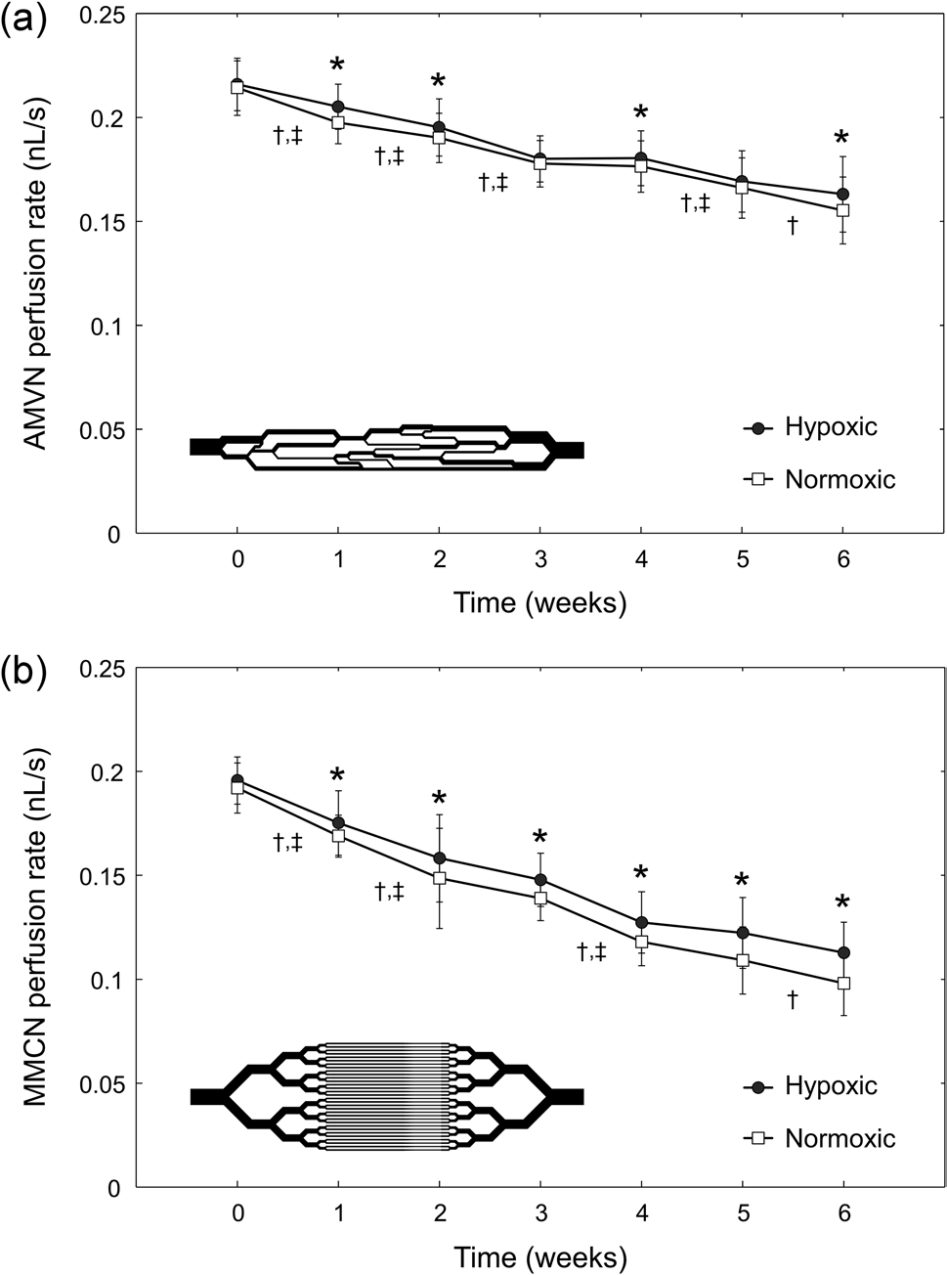
The change in **(a)** AMVN and **(b)** MMCN perfusion rates for RBCs stored under normoxic and hypoxic conditions over the course of 6-week hypothermic storage. Values shown are mean ± standard deviation (n = 9). Statistically significant differences (*p* < 0.05) between hypoxically and normoxically stored RBCs of the same storage duration are marked by an asterisk (*). Statistically significant differences (*p* < 0.05) between subsequent weeks of storage are marked by a dagger (†) for normoxically stored RBCs and a double dagger (‡) for hypoxically stored RBCs.

Figure 3 shows the progressive decline of the AMVN and MMCN perfusion rates over the course of 6-week hypothermic storage for both storage conditions, however perfusion rates were consistently higher for hypoxic than for normoxic storage. For normoxRBCs the AMVN perfusion rate decreased by 27.5% between week 0 and week 6 of storage, while for hypoxRBCs the AMVN perfusion rate decreased by 24.5% (Fig. 3a). At the beginning of storage, there was no statistically significant difference in AMVN perfusion rate between the two storage conditions, but after 1 week of storage the AMVN perfusion rates diverged and hypoxRBCs perfused the AMVN significantly faster than normoxRBCs at weeks 1, 2, 4 and 6. At the end of storage, the AMVN perfusion rate for hypoxRBCs was 5.0% higher than for normoxRBCs. A statistically significant decrease in AMVN perfusion rate compared to week 0 was observed after only 1 week of storage for both storage conditions.

Similarly, the MMCN perfusion rate for normoxRBCs decreased by 49.0% between week 0 and week 6, while for hypoxRBCs the MMCN perfusion rate decreased by 42.4% (Fig. 3b). At the beginning of storage, there was no statistically significant difference in MMCN perfusion rate between normoxic and hypoxic storage, but after 1 week of storage the MMCN perfusion rates diverged and hypoxRBCs perfused the MMCN significantly faster than normoxRBCs for the remainder of the storage duration. At the end of storage, the MMCN perfusion rate for hypoxRBCs was 15.0% higher than for normoxRBCs. A statistically significant decrease in MMCN perfusion rate compared to week 0 was observed after only 1 week of storage for both storage conditions.

### Capillary plugging in the microfluidic networks

**Figure 4:**
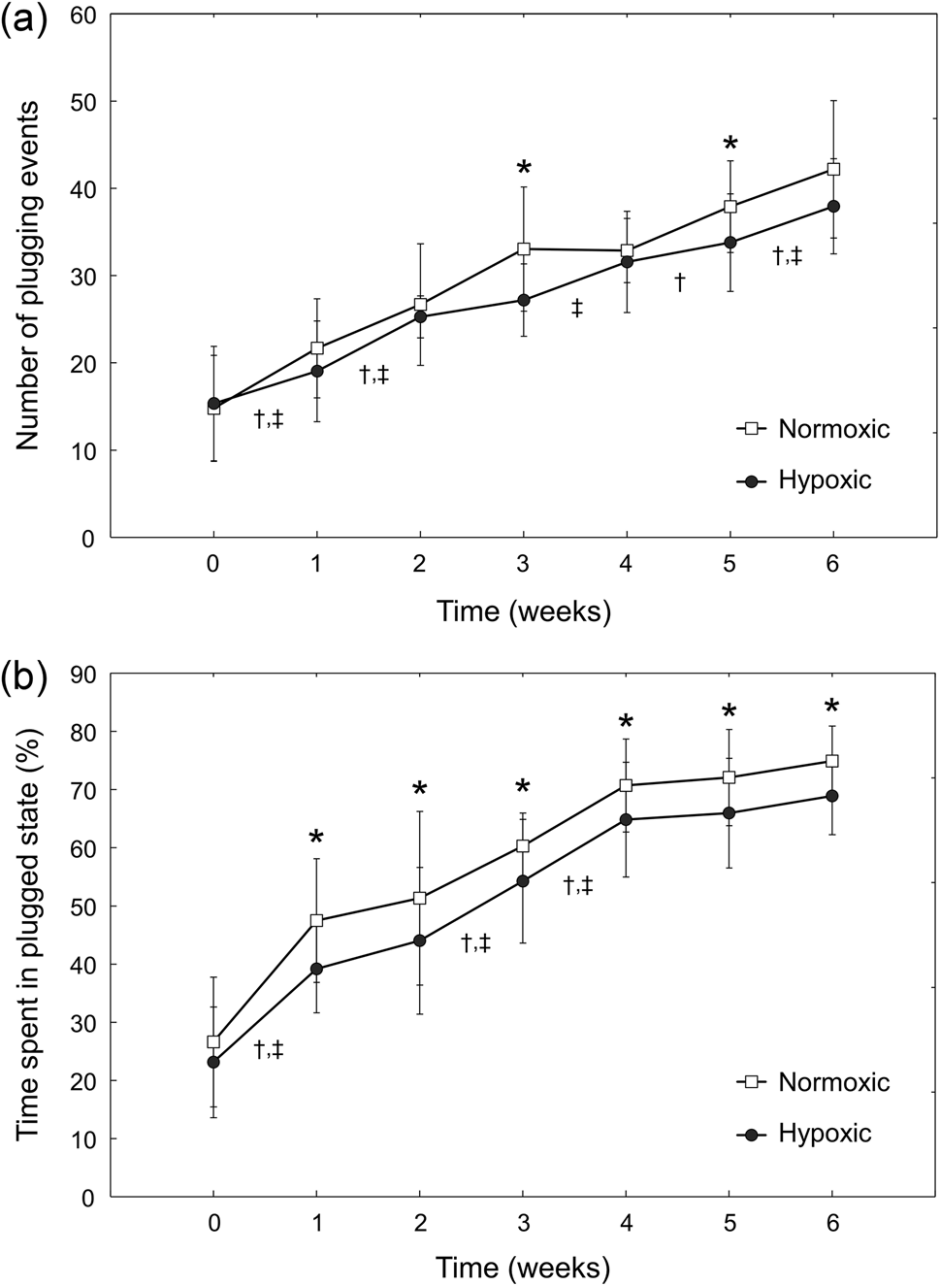
Intermittent plugging of individual MMCN microcapillaries by RBCs stored under normoxic and hypoxic storage conditions. (**a**) Number of ‘plugging’ events; a ‘plugging’ event was defined as a decrease in microcapillary RBC velocity below a baseline value (baseline velocity = mean RBC velocity on week 0—two standard deviations). (**b**) Total percent of observation time all MMCN microcapillaries spent in a plugged state (i.e. percent of time microcapillaries operated below baseline velocity). Values shown are mean ± standard deviation (n = 9 RBC units). Statistically significant differences (*p* < 0.05) between normoxic and hypoxic storage of the same duration are marked by an asterisk (*). Statistically significant differences (*p* < 0.05) between subsequent weeks of storage are marked by a dagger (†) for normoxic, and a double dagger (‡) for hypoxic storage.

We measured the flow rate of RBCs passing through the each of the 32 microcapillaries comprising the MMCN in order to determine the number of times individual poorly deformable RBCs intermittently ‘plugged’ a capillary before passing through the network (Fig. 4a), and the total percentage of the observation time any of the capillaries spent in the ‘plugged’ state (Fig. 4b). Overall, the number of MMCN plugging events as well as the percentage of time MMCN capillaries spent in a plugged state increased progressively throughout storage for both types of storage. The number of MMCN plugging events and percentage of time MMCN capillaries spent in a plugged state was generally lower for hypoxRBCs than for normoxRBCs. The mean number of plugging events increased 2.9-fold by week 6 for normoxRBCs, but only 2.5-fold for hypoxRBCs. A statistically significant increase in the number of MMCN plugging events compared to week 0 was observed after 1 week of storage for normoxic storage, and after 2 weeks for hypoxic storage. From week 1 onwards, the MMCN capillaries spent significantly more time in the plugged state for normoxic than for hypoxic storage (an 8.0% difference by the end of storage). A statistically significant increase in the percentage of time the MMCN capillaries spent in a plugged state compared to week 0 was observed after 1 week for both storage conditions.

### The change of RBC morphology during storage

**Figure 5:**
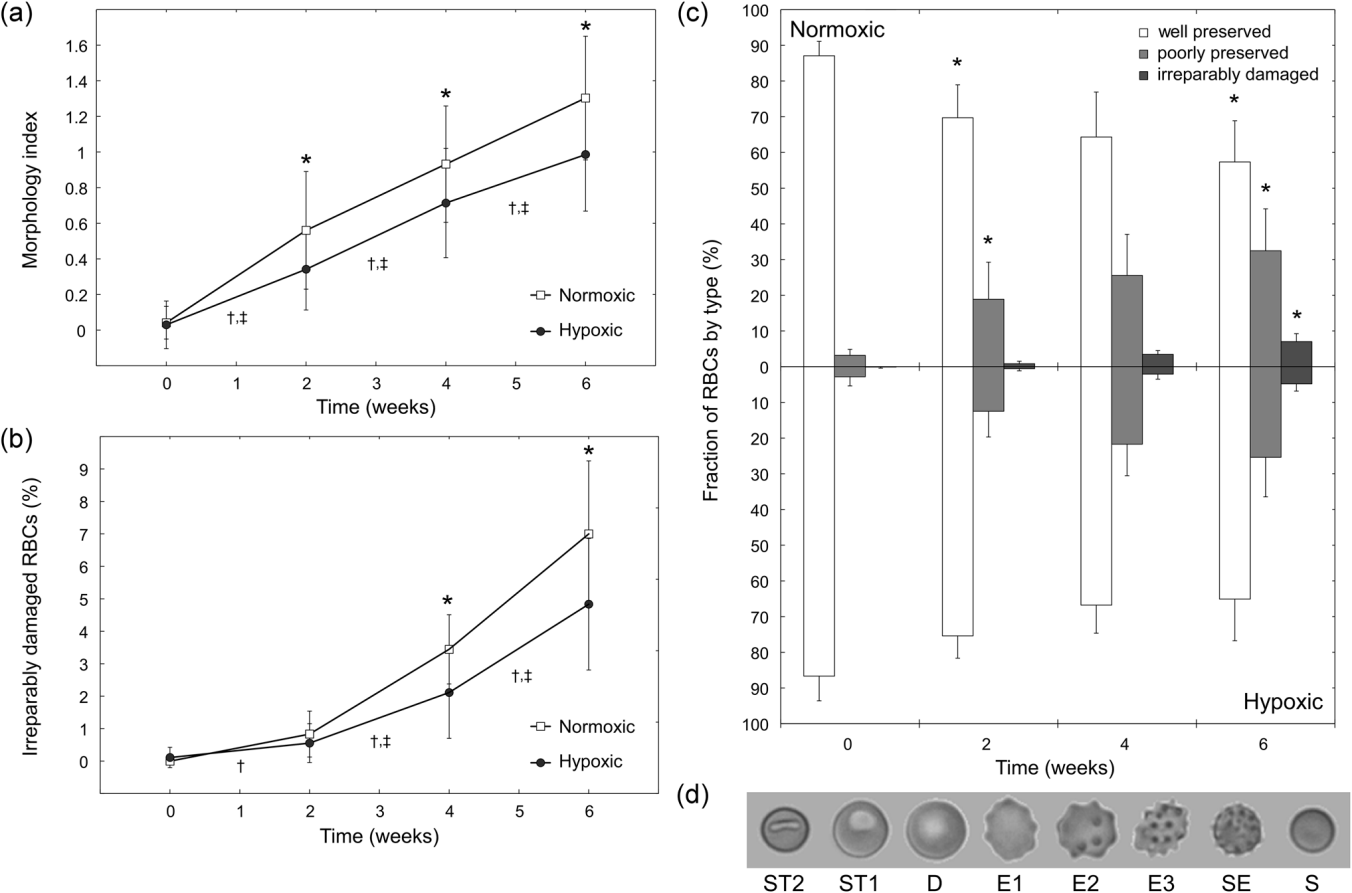
Deterioration of RBC morphology during 6-week hypothermic storage under normoxic and hypoxic conditions. (**a**) Overall morphology index (MI). (**b**) The fraction of ‘irreparably damaged’ RBCs (SE and S) in the overall population of stored RBCs. (**c**) The change in distribution of all RBC within a unit among well-preserved (D and E1, white bar), poorly-preserved (E2 and E3, gray bar) and irreparably damaged (SE and S, dark gray bar) fractions in each unit. Values shown are mean ± standard deviation (n = 9 RBC units). Statistically significant differences (*p* < 0.05) between normoxic and hypoxic storage of the same duration are marked by an asterisk (*). Statistically significant differences (*p* < 0.05) between subsequent weeks of storage are marked by a dagger (†) for normoxic, and a double dagger (‡) for hypoxic storage. (**d**) Representative images of RBC morphologies: discocyte (D), echinocyte I (E1), echinocyte II (E2), echinocyte III (E3), sphero-echinocyte (SE), spherocyte (S), stomatocyte I (ST1) and stomatocyte II (ST2).

Figure 5 illustrates the change in RBC morphology over the course of 6-week hypothermic storage. RBCs were classified as well-preserved (D and E1), poorly-preserved (E2 and E3), or irreparably damaged (SE and S) cells^33^. Overall, the fraction of well-preserved RBCs decreased and the fractions of poorly-preserved and irreparably damaged RBCs increased over time for both types of storage (Fig. 5c). The morphology index (MI) for normoxRBCs was significantly higher than for hypoxRBCs starting on week 2 (Fig. 5a). A statistically significant increase in MI compared to week 0 occurred after 2 weeks for both types of storage. Importantly, there was a statistically significantly lower percentage of irreparably damaged (spherical) hypoxRBCs than normoxRBCs by week 6 (Fig. 5b). A statistically significant increase in the percentage of irreparably damaged RBCs compared to week 0 occurred after 2 weeks for normoxic, and after 4 weeks for hypoxic storage.

## Discussion

Normal RBCs are biconcave discs with high membrane area to volume ratio, which allows them to deform sufficiently to pass through capillaries narrower than the cell diameter^13,33^. Numerous adverse changes in RBC mechanical and biochemical properties occur during ex vivo hypothermic storage^4,5^, and oxidative damage to cell membrane and cytoskeleton is one of the key causes of these storage lesions^34–36^. While in storage, RBCs undergo echinocytosis, becoming increasingly spicular and progressively more spherical (due to the loss of the excess membrane area via vesiculation)^33–35^. These changes lead to a gradual decline of the ability of stored RBCs to deform and traverse capillaries^37^. The susceptibility of individual RBCs to storage-induced deterioration varies greatly even within the same unit, resulting in a highly heterogeneous RBC population after only days of storage^13^. Interestingly, the morphology of stored RBCs can be partially restored by washing, except for SE and S cells which appear to be damaged irreversibly^38^. In this study we hypothesized that presence of even a relatively small subpopulation of such irreversibly-damaged, non-deformable RBCs in a sample would result in a significant impairment of its overall rheological function (perfusion of capillary networks), but not the standard population-average deformability values produced by ektacytometry (elongation under shear in a hyperviscous media).

Our findings largely support this hypothesis. Indeed, the decreases in EI measured by RheoScan-D and LORRCA between week 0 and week 6 of storage were only 5.8–7.1% and 5.4–7.0%, respectively (Fig. 2). However, over the same storage period bulk AMVN perfusion rate decreased by 24.5–27.5% (Fig. 3a), bulk MMCN perfusion rate decreased by 42.4–49.0% (Fig. 3b), number of plugging events increased by 147.3–185.5% (Fig. 4a), and time MMCN capillaries spent in plugged state increased by 181.4–197.7% (Fig. 4b). The drastically larger changes in AMVN and MMCN device measurements as compared to ektacytometry measurements performed on RBCs from the same units suggest that the microfluidic devices are more sensitive to storage-induced changes in RBC rheological properties. This higher sensitivity of the MMCN to changes in deformability was also evident in a recent study that compared MMCN perfusion rate to RBC filterability^27^. Additionally, the number of statistically significant changes between subsequent weeks of storage was much lower for RheoScan-D (1 of 6 weeks for normoxic and 1 of 6 weeks for hypoxic storage at 3 Pa; 1 of 6 weeks for normoxic and 3 of 6 weeks for hypoxic storage at 17 Pa; Fig. 2a) and LORRCA (1 of 6 weeks for both normoxic and hypoxic storage at 3 Pa; 2 of 6 weeks for normoxic and 1 of 6 weeks for hypoxic storage at 16.87 Pa; Fig. 2b) compared to AMVN perfusion rate (5 of 6 weeks for normoxic and 4 of 6 weeks for hypoxic storage; Fig. 3a), MMCN perfusion rate (4 of 6 weeks for normoxic and 3 of 6 weeks for hypoxic storage; Fig. 3b), MMCN plugging events (4 of 6 weeks for both normoxic and hypoxic storage; Fig. 4a) and time MMCN spent plugged (3 of 6 weeks for both normoxic and hypoxic storage; Fig. 4b). These data suggest that microfluidic capillary networks are better than ektacytometry at resolving changes in RBC deformability that occur over relatively short periods of hypothermic storage.

Interestingly, the deformability measurements made by both ektacytometers indicated no consistent, statistically significant difference between RBCs stored under normoxic and hypoxic conditions (Fig. 2). One possible explanation is that the irreversibly-damaged, non-deformable RBCs made up too small of a fraction of the overall RBC population to affect the laser diffraction patterns. After 6 weeks of storage, 7.0 ± 2.2% of normoxRBCs and 4.8 ± 2.0% of hypoxRBCs were damaged irreversibly (Fig. 5b). The EI measurements were unable to detect this difference between the two groups of samples. In contrast, the overall rate at which hypoxRBCs were able to perfuse the two distinct microfluidic capillary networks was consistently faster than that of normoxRBCs (Fig. 3). These data suggest that even a relatively small subpopulation of poorly deformable RBCs could have a profound impact on the functional properties of a RBC unit as whole. Hypoxic storage appears to decrease the fraction of poorly deformable RBCs by minimizing oxidative damage experienced by RBC membrane and cytoskeleton over the course of 6-week hypothermic storage^11^. This conclusion was supported by the lower number of plugging events and lower percentage of time spent in a plugged state by MMCN microcapillaries (Fig. 4). This conclusion was further supported by direct morphological analysis, which found that the MI of hypoxRBC units was consistently better than that of normoxRBC units, largely because the fractions of poorly-preserved and irreparably-damaged RBCs were consistently smaller in the case of hypoxic storage (Fig. 5a).

The AMVN has been used extensively to study the effect of various rheological properties of blood on the dynamics of blood flow in capillary networks^16,17,37,39,40^. However, the AMVN has several important limitations: (1) it is constructed of PDMS whose mechanical and biochemical properties are rather different from that of real blood vessels^41^, (2) the capillary microchannels have rectangular cross-sections which may affect certain aspects of cellular traffic in the network^42^, and (3) the network topology is dramatically simplified relative to the real microvasculature^43^. The MMCN shares limitations (1) and (2), while also intentionally exaggerating certain aspects of the real microvasculature^17,27^. In their current forms, the AMVN and MMCN devices have utility as research tools but require prohibitive amounts of hands-on user input (e.g. manually focusing microscope on microchannels; manually adjusting water column to control driving pressure) to be deployed in a time-sensitive, high-throughput setting. Another limitation of this study was that all RBC units were removed from the blood bank refrigerator, mixed and sampled weekly throughout storage. These extra manipulations might have resulted in unknown changes to RBC properties compared to undisturbed units. However, because the sampling procedure was identical for all units stored under both normoxic and hypoxic conditions, these potential changes are unlikely to have affected the outcomes of this study significantly.

In a previous study, relative deformability measured by MMCN showed moderate correlation with autologous 24-h post transfusion recovery for normoxically stored RBCs after 6 weeks (Pearson’s R = 0.475, *p* < 0.03; 21 subjects) but not for hypoxically stored RBCs (Pearson’s R = 0.024, *p* > 0.9; 19 subjects). Although the correlation was not strong, it was highest among numerous parameters examined, and it was closely followed by ATP (R = 0.442, *p* < 0.036)^44^. If the sensitivity of MMCN can be further increased to yield high correlation with 24-h post transfusion recovery, it could be used as an objective, functional, physiologically-focused test of the quality of RBC units used for transfusion suspected of having compromised 24-h recovery, to reduce potential clinical sequelae of transfusion. Further improvements in the designs of the AMVN and MMCN devices and deployment in future clinical studies are planned.

While the net result of most RBC transfusions is undoubtedly beneficial, infusion of cells that had accumulated irreversible damage during storage and lost their ability to deform sufficiently to traverse the capillary networks has no therapeutic benefit and may even be harmful to some patients. Our in vitro results suggest that irreparably damaged, poorly deformable RBCs within otherwise well-preserved RBC units may significantly alter microvascular blood flow in vivo and could therefore contribute to differential patient outcomes. Even if microcapillary networks in vivo are better able to deal with poorly deformable RBCs (e.g. via biochemically regulated vasomotion) than the comparatively rigid PDMS microfluidic devices, the impact of these damaged RBCs on the overall rheology of stored blood remains clinically significant^37^. Poorly deformable, irreparably damaged RBCs most likely constitute the majority of the RBCs which are known to not survive beyond the 24 h immediately after transfusion^7,45^. RBCs which lyse or are removed from circulation via sequestration by the spleen soon after transfusion do not serve their intended purpose of microvascular perfusion and tissue oxygenation, but instead contribute to negative side effects of transfusion (e.g. iron overload)^18^. It would therefore be beneficial for methods of measuring RBC deformability to be sufficiently sensitive to detect subpopulations of irreparably damaged, poorly deformable within otherwise well-preserved stored RBC units prior to transfusion.

## Conclusions

Here we showed that small subpopulations of irreparably damaged, poorly deformable RBCs significantly decrease the ability of entire populations of stored RBCs to traverse microfluidic networks and significantly increase the occurrence and duration of RBCs plugging events. This was because flow through the AMVN and MMCN microfluidic networks was dependent on the ability of individual RBCs to deform and pass through narrow constrictions smaller than the resting diameter of the RBCs. We also showed that subpopulations of poorly deformable RBCs do not significantly impact the measurements by conventional ektacytometers. This was likely because the diffraction patterns used to calculate elongation index were generated by the entire population of RBCs, and the effect of the relatively small fraction of poorly deformable cells was masked by the much stronger signal produced by well-preserved RBCs. Because AMVN and MMCN measurements showed larger percent changes over the course of 6-week hypothermic storage as well as a larger difference between normoxic and hypoxic conditions, we conclude that microfluidic networks are more sensitive tools for measuring RBC deformability than ektacytometry.
